# Supplementary material for: Novel Therapeutic Effects of Pterosin B on Ang II-Induced Cardiomyocyte Hypertrophy
Source: Molecules. 2020 Nov 12;25(22):5279. doi: 10.3390/molecules25225279 (PMC7697794; doi:10.3390/molecules25225279)

Novel therapeutic effects of Pterosin B on Ang II-induced cardiomyocyte hypertrophy

Chang Youn Lee[1,#], Han Ki Park[2,#], Bok-Sim Lee[3,#], Seongtae Jeong[4], Sung-Ae Hyun[5], Jung-Won Choi[6], Sang Woo Kim[7], Seahyoung Lee[8], Soyeon Lim[9,*], Ki-Chul Hwang[10,*]

[1] Substance Abuse Pharmacology Group, Korea Institute of Toxicology, KRICT, Daejeon 34114, Republic of Korea; changyeon.lee@kitox.re.kr

[2] Division of Cardiovascular Surgery, Department of Thoracic and Cardiovascular Surgery, Severance Cardiovascular Hospital, Yonsei University College of Medicine, Seoul, Korea; HANK@yuhs.ac

[3] Institute for Bio-Medical Convergence, College of Medicine, Catholic Kwandong University, Gangneung-si, Gangwon-do 210-701, Korea; alsgur04@nate.com

[4] Institute for Bio-Medical Convergence, College of Medicine, Catholic Kwandong University, Gangneung-si, Gangwon-do 210-701, Korea ; 91seongtae@gmail.com

[5] Substance Abuse Pharmacology Group, Korea Institute of Toxicology, KRICT, Daejeon 34114, Republic of Korea; sahyun@kitox.re.kr

[6] Institute for Bio-Medical Convergence, College of Medicine, Catholic Kwandong University, Gangneung-si, Gangwon-do 210-701, Korea; jungwonjian@gmail.com

[7] Institute for Bio-Medical Convergence, College of Medicine, Catholic Kwandong University, Gangneung-si, Gangwon-do 210-701, Korea ; doctor7408@gmail.com

[8] Institute for Bio-Medical Convergence, College of Medicine, Catholic Kwandong University, Gangneung-si, Gangwon-do 210-701, Korea ; sam1017@ish.ac.kr

[9] Institute for Bio-Medical Convergence, College of Medicine, Catholic Kwandong University, Gangneung-si, Gangwon-do 210-701, Korea ; slim724@cku.ac.kr

[10] Institute for Bio-Medical Convergence, College of Medicine, Catholic Kwandong University, Gangneung-si, Gangwon-do 210-701, Korea ; kchwang@cku.ac.kr

# Chang Youn Lee, Han Ki Park, and Bok-Sim Lee contributed equally to the work.

* Correspondence: slim724@cku.ac.kr; Tel.: +82-32-290-2777 (S.L.), kchwang@cku.ac.kr; Tel.: +82-32-290-2774 (K-C.H.)

Supporting information

Materials and Methods

Measuring AGE-RAGE interaction in vitro

To investigate the interaction of RAGE-Pterosin B in vitro, CircuLex AGE-RAGE in vitro Binding Assay Kit (CircuLex™, cat#: CY-8151, MBL Life science, Japan) was used according to user's manuals. Briefly, various concentration of Pterosin B (test compound), vehicle control, and inhibitor control were added into microtiter wells of AGE2-BSA coated microplate with His-tagged sRAGE, and then incubated at room temperature for 60 min. After 4-times washing step, HRP conjugated anti-His-tag antibody was added into each well and incubated at room temperature for 60 min. Next, chromogenic substrate (tetra methylbenzidine) was added after 4-times of washing and wells were incubated at room temperature for 5-15 min. Finally, absorbance in each well was measured using a spectrophotometric plate reader (Multiskan™ microplate photometer, ThermoFisher Scientific, MA, USA) at dual wavelengths of 450/540nm after adding stop reagent.

Whole-cell voltage-clamp recordings

In order to evaluate the effect on the cardiac single ion channel, cells with permanently expressed ion channels were used. The cell lines were obtained from B'SYS GmbH (Witterswil, Switzerlznd). CHO hERG cells were cultured according to the manufacturer's instructions. At least 7 days prior to use, a vial of frozen cells was thawed. Cells were incubated at 37°C in a 5% CO_2 atmosphere as a monolayer and then sub-cultured twice-weekly. The external solution for recording the IKr channel currents was normal Tyrode (NT) solutions as follows (in mM) : 143 NaCl, 5.4 KCl, 5 HEPES, 0.33 NaH_2PO_3, 1.8 $CaCl_2$, 0.5 $MgCl_2$ and 10.0 glucose (pH adjusted to 7.4 with NaOH). The internal solution for IKr contained the following (in mM): 130 KCl, 5 EGTA, 10 HEPES, 1 $MgCl_2$, 5 Mg-ATP (pH adjusted 7.25 with KOH).

Results

Supplementary Figures

Figure S1. Binding ability of Pterosin B on RAGE. Inhibition rate of Pterosin B was converted based on vehicle control (inhibition rate of vehicle control=0%). ***p<0.001. n=3.

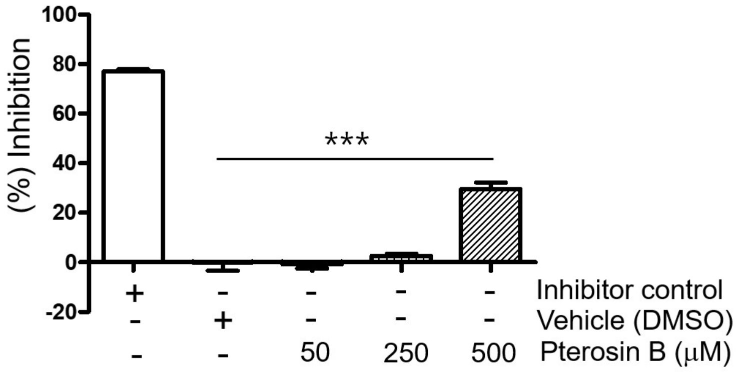

Figure S2. The effect of Pterosin B on cardiac ion channel current. (A) Representative traces demonstrating the dose-dependent effect of Pterosin B on hERG current expressed in CHO hERG cells. (B) The hERG channel inhibition rate according to the pterosin B concentration was quantified. Control vs 50μM Pterosin B (*p<0.05). n=3.

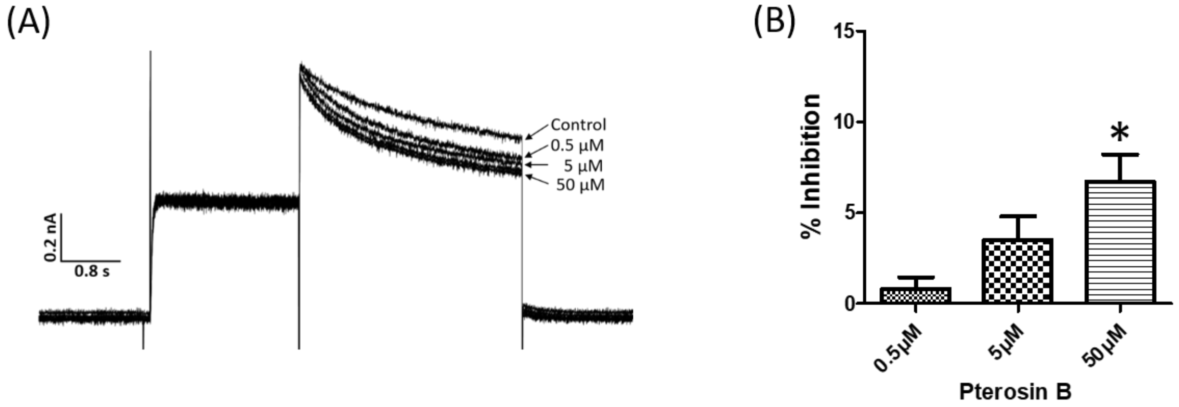

Figure S3. Raw data for Figures

1) Raw data for Figure 2A

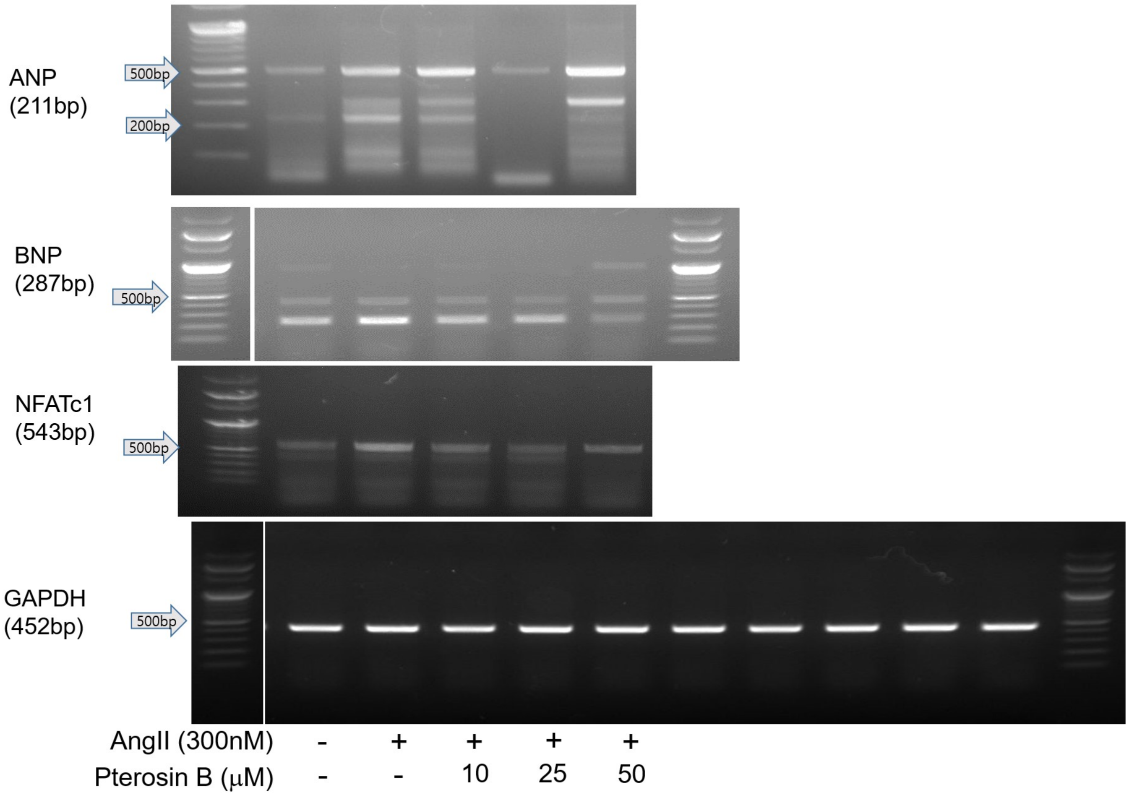

2) Raw data for Figure 3A

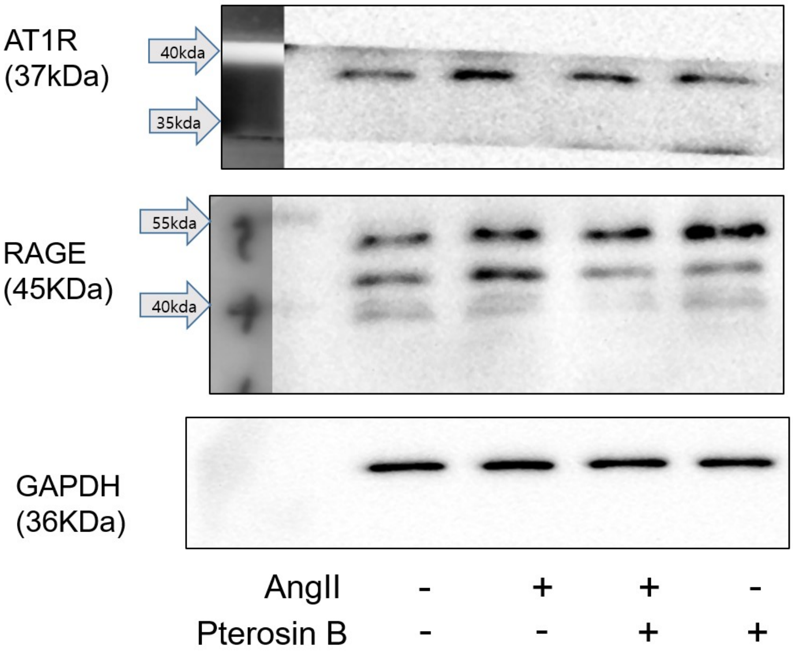

3) **Raw data for Figure 3D**

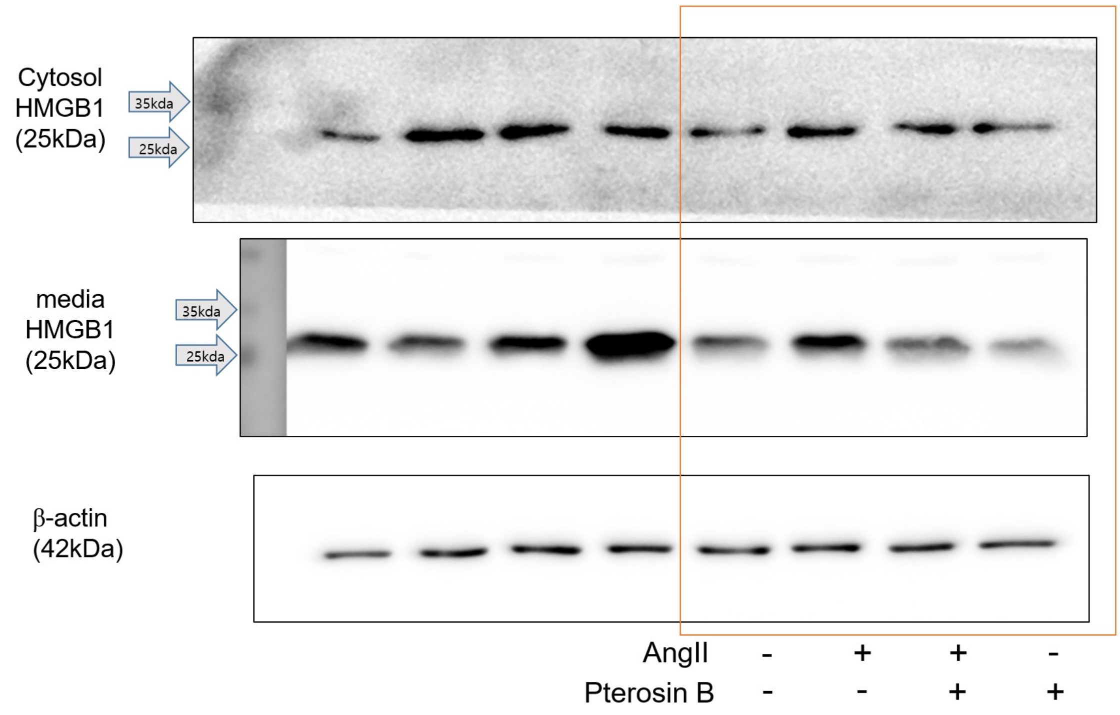

4) **Raw data for Figure 3F**

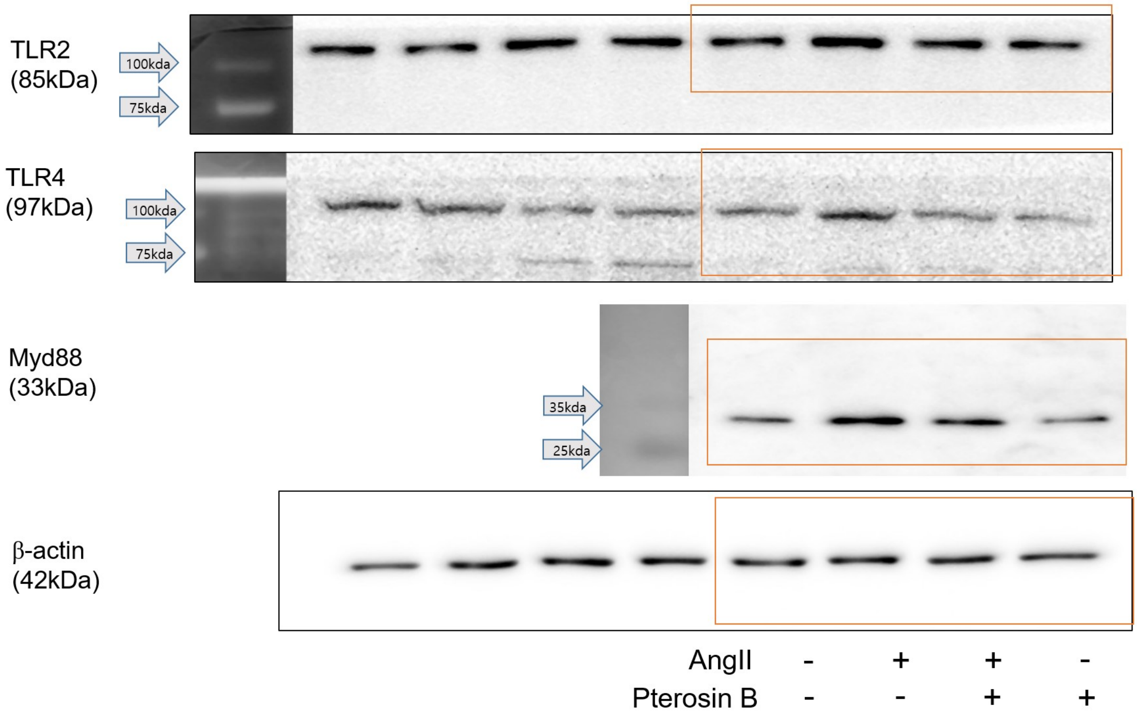

5) Raw data for Figure 4A

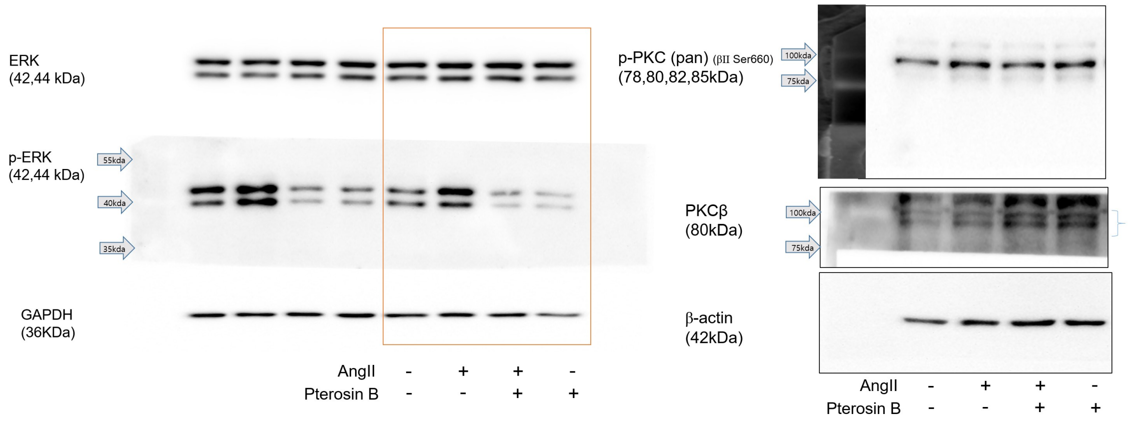

6) Raw data for Figure 4C

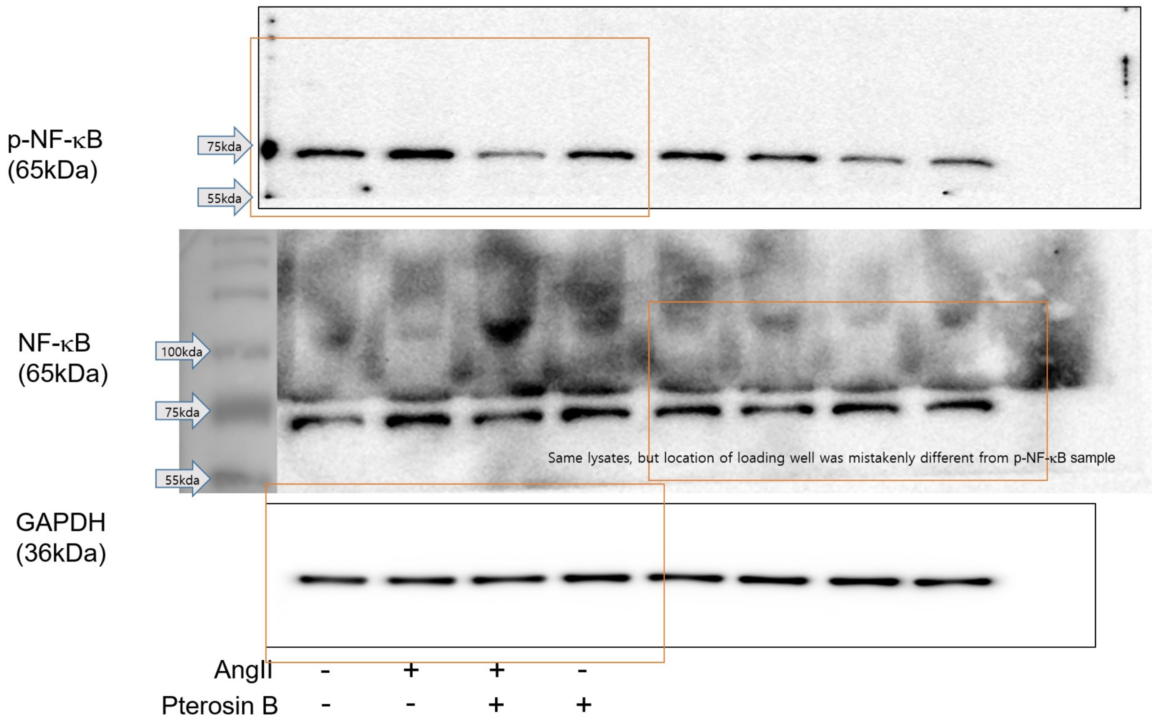

7) Raw data for Figure 5C

WB data in main article (left) → To confirm size marker for NOXs WB, we ran SDS gel again (right)

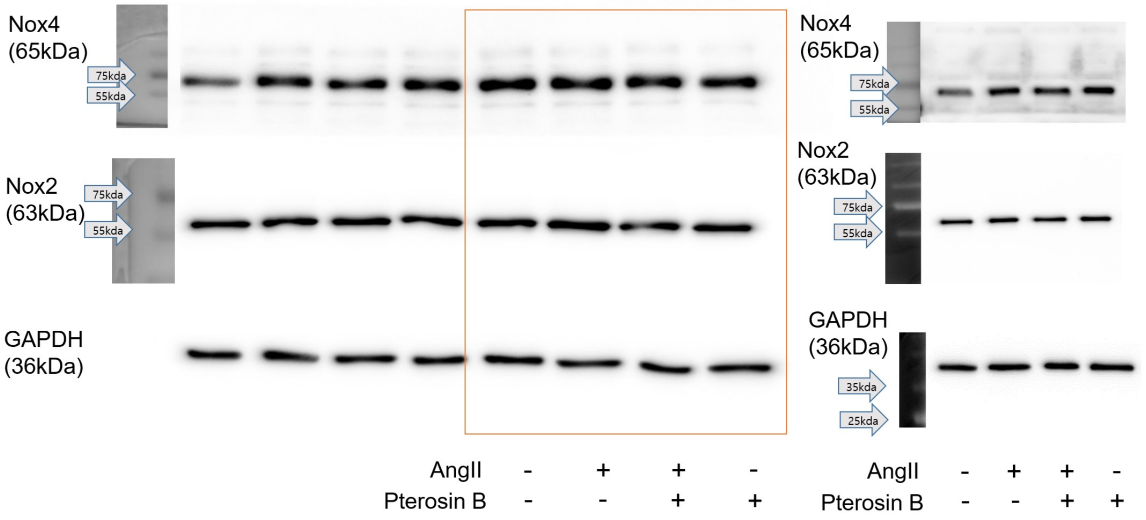

Supplement: Supplementary file 1 [file molecules-25-05279-s001.pdf]
